# A Cross-Sectional Study of the Relationship Between Perceived Stress and Thyroid Function Among Apparently Normal Women in the Reproductive Age

**DOI:** 10.7759/cureus.55567

**Published:** 2024-03-05

**Authors:** Sandhya H Puttaswamy, Neeta P Nandibewur, Pawan Kumar, Vijay Venkataiah, Mohammed Jaffer Pinjar

**Affiliations:** 1 Department of Physiology, Vijayanagar Institute of Medical Sciences, Ballari, IND; 2 Department of Community Medicine, Vijayanagar Institute of Medical Sciences, Ballari, IND; 3 Department of Biochemistry, Vijayanagar Institute of Medical Sciences, Ballari, IND; 4 Department of Physiology, All India Institute of Medical Sciences, Deoghar, IND

**Keywords:** tsh, total t4, total t3, subclinical hypothyroidism, perceived stress

## Abstract

Background: A substantial majority of women in India report experiencing stress frequently, with a significant number indicating a lack of time for relaxation. Women within a central productive age bracket often report higher stress levels. Chronic stress can lead to the development of autoimmune disorders affecting the thyroid gland.

Objective: To evaluate the relationship between perceived stress and thyroid function among apparently normal women of reproductive age.

Materials and methods: The present study was conducted at the Vijayanagar Institute of Medical Sciences (VIMS) after obtaining clearance from the Institutional Ethical Committee and informed written consent from the participants. One hundred and fourteen working women aged 20-49 who consented to the study and had no personal or family history of medical illness or thyroid disease were randomly selected. Stress levels were measured using a Perceived Stress Scale (PSS), and thyroid parameters (total triiodothyronine [T3], total thyroxine [T4], and thyroid-stimulating hormone [TSH]) in blood samples were assessed by the electrochemical luminescence immunoassay method. Anthropometric parameters such as age and body mass index (BMI), as well as vital parameters like pulse rate and blood pressure, were measured for all participants. A detailed history was also recorded, including marital status, duration of married life, education, number of children, type of family, per capita income, phase of menstrual cycle, and dietary habits.

Results: The Statistical Package for the Social Sciences (SPSS) software version 22.0 was used for statistical analysis. The analysis used Pearson's chi-square test, Student's t-test, and binary logistic regression. A positive correlation was observed between PSS and TSH (correlation coefficient "r" value = 0.060) without a significant p-value. Participants were divided into two groups based on TSH values: those with normal thyroid function (TSH <4.2 international units [IU]) and those with subclinical hypothyroidism (SCH) (TSH >4.2 IU). Both groups had total T3 and T4 levels within the normal reference range. A highly significant difference was observed for age, BMI, TSH, marital status, and duration of married life between women with normal thyroid function and those with SCH. No significant difference was found between the two groups for PSS.

Discussion: Both acute and chronic stress affect thyroid function through the hypothalamus-pituitary-thyroid (HPT) axis and the hypothalamus-pituitary-adrenal (HPA) axis. Psychological and physiological stressors induce immune modulations that can lead to autoimmune thyroid diseases, resulting in hypothyroidism.

Conclusion: The study examined the link between stress and thyroid health in women of childbearing age, revealing a trend where higher stress levels corresponded with increased TSH levels, though not significantly. It also found that older age, higher BMI, and longer duration of marriage were linked to a greater occurrence of SCH. These findings underscore the potential influence of lifestyle factors and stress on thyroid function, suggesting that stress management and demographic factors should be considered in managing thyroid health. For women of reproductive age under high stress, routine monitoring of thyroid function could be beneficial for overall health maintenance.

## Introduction

Stress is the body’s response to the daily events that occur in life. Women of reproductive age often experience heightened stress due to the multitude of roles they assume, which include family obligations, caregiving for children and elderly parents, and managing work responsibilities, among other duties. Apart from these, the other causes of stress for women are financial issues, job security, health, and relationship issues in the present highly competitive metropolitan culture. Stress can be positive and motivate women to achieve notable goals. However, stress can also be negative and destructive, taking its toll in many life areas. As the demands increase to fulfill multiple roles for women as a collective social well-being, they may feel overwhelmed with time pressure and unmet obligations. The accumulation of these responsibilities may lead to chronic stress over time. Recent studies have shown that about 42 million people in India suffer from thyroid diseases. Chronic stress cannot only create havoc on overall health and well-being but can also affect the functioning of the thyroid gland [1].

The thyroid gland works in tandem with the adrenal gland, which releases cortisol to enhance various bodily functions when encountering a small amount of stress. Recent data suggests that stress can be one of the environmental factors for the development of autoimmune thyroid diseases such as Grave's disease and Hashimoto's thyroiditis [2]. So stress, the nonspecific result of any demand on the body, affects the hypothalamus-pituitary-adrenal (HPA) axis system, which also maintains homeostasis. Stressors or stressful circumstances influence the hypothalamus-pituitary-thyroid (HPT) axis and psychological and physiological responses [3].

Literature suggests that acute stress can lead to a decrease in the secretion of thyroid-stimulating hormone (TSH) and a decrease in total triiodothyronine (T3). These changes are produced by the glucocorticoids released by the adrenal glands that affect the HPT axis and are mediated at the level of the hypothalamus. Also, there is a strong correlation between plasma T3 levels and the activity of the central dopamine and serotonin system. Individual differences in thyroid hormone levels might also predict vulnerability to psychiatric diseases [4]. Some studies have established a link between thyroid function and depression. These studies indicate an association of depression with subtle thyroid abnormalities [5,6]. Recently conducted studies have indicated a significant positive correlation between perceived stress and serum prolactin levels in subclinical and newly diagnosed cases of hypothyroidism [7,8]. Women of reproductive age feel stressed most of the time with no time to relax because of the multiple roles that they take on in this modern, competitive world. Eighty-seven percent of Indian women feel stressed most of the time, with 82% having no time to relax [1].

Studies have been done in the recent past to find out the correlation between perceived stress and thyroid function among women of reproductive age who were newly diagnosed with hypothyroidism. However, the present study aims to determine the relationship between perceived stress and the function of the thyroid gland among apparently normal women of reproductive age. Providing stress-relieving tips to women, such as enrolling in stress management programs or making lifestyle modifications, may improve their quality of life. Data about the relationship between thyroid function and perceived stress is lacking in the South Indian population; hence, this study was taken up.

## Materials and methods

This study was conducted on 114 apparently normal working women of reproductive age (20-49 years) at Vijayanagar Institute of Medical Sciences (VIMS) after obtaining institutional ethical clearance in 12 months. Informed written consent was obtained from all the participants. The formula used to calculate the sample size was as follows: n = [{ (Z1 + Z2)/ Cr}2 +3] and Cr = ½ [LOGe (1 + r) / (1 - r)], where “Cr” and “r” are the correlation coefficients, n is the minimum sample size, and Z1 and Z2 are the Z values associated with alpha and beta, respectively.

Inclusion criteria

Apparently normal working women of reproductive age (20-49 years) willing to participate in the study were included.

Exclusion criteria

The study excluded women who had either a personal or family history of thyroid diseases to avoid confounding genetic influences. Additionally, those diagnosed with other medical conditions, such as diabetes, hypertension, asthma, autoimmune diseases, liver or renal diseases, as well as seizures, were not included in the research to isolate the impact of stress on thyroid function. Women who were experiencing psychiatric conditions like depression and anxiety were also excluded to eliminate the potential overlapping effects of these conditions on stress levels. Moreover, the study did not consider pregnant, lactating, or postmenopausal women, as these physiological states could independently affect thyroid function. Finally, women with a history of smoking, alcoholism, or drug use were omitted from the study to avoid the confounding effects of these substances on both stress and thyroid function.

In this study, a comprehensive set of parameters was examined to assess the impact of stress on thyroid function. Anthropometric measurements were taken, including the height and weight of participants, from which body mass index (BMI) was calculated. Vital parameters such as pulse rate and blood pressure were also meticulously measured to provide a baseline for each participant's health status. To gauge the psychological aspect, Perceived Stress Scale (PSS) scores were obtained, reflecting the participants' subjective experience of stress. On the physiological front, thyroid function was evaluated by measuring levels of TSH, T3, and total thyroxine (T4) in the blood, providing a comprehensive view of the endocrine system's response to stress.

Participants were recruited by random sampling. Participants were screened based on the inclusion and exclusion criteria before their recruitment in the study. The phase of the menstrual cycle of all the participants was recorded, and the majority of the participants were in the post-menstrual phase. Apart from the above parameters, the history of the following was recorded: the educational qualification, number of years of married life, number of kids, types of family, whether nuclear or joint, and per capita income of the family. Most participants were postgraduates, married with two kids in the nuclear family, and of moderate per capita income. Most participants were non-vegetarian, and their dietary habits were similar. Two milliliters of blood sample was drawn from each of the 114 participants using a sterile needle under the aseptic precautions. Then, the blood sample was sent to the (central laboratory of VIMS) biochemistry laboratory in sterile vials for further analysis.

The PSS, which is a precise measure of personal stress, was used to assess the stress levels among the participants. PSS is a questionnaire having 10 questions that help us understand how different situations affect our feelings and our perceived stress. The questions in this scale are about our feelings and thoughts during the last month. The responses to each question are graded from 0 to 4 (0 - never, 1 - almost never, 2 - sometimes, 3 - fairly often, 4 - very often). Then, the total PSS score is added for all 10 questions to get the total score. Based on the PSS score, stress perceived by the participants was graded as low stress: 0-13, moderate stress: 14-26, and severe stress: 27-40 [9,10]. The electro-chemiluminescent-immune assay method was used to assess the total T3, total T4, and TSH levels in the participants' blood samples in the biochemical laboratory of VIMS, Ballari.

Statistical analysis

The computer software SPSS version 22.0 (IBM Corp, Armonk, NY) was used to analyze the data. Jamovi version 2.3 (Jamovi, Sydney, Australia) was also used to analyze the data. Chi-square and t-test were the statistical methods used, and binary logistic regression was the only statistical model used to analyze the data. A p-value <0.05 implies significance and a p-value <0.01 was said to be highly significant.

## Results

After analyzing the blood samples among 114 normal women in the reproductive age group (20-49 years), it was found that 18.4% had SCH and 81.6% had normal TSH. A TSH value of 4.2 microIU/mL is said to be normal and above what was considered hypothyroidism [11]. Among 114 participants, 17.5% had a low PSS score, 78.9% had a moderate PSS score, and 3.5% had a high PSS score (Table 1). The Pearson correlation between TSH and PSS was positive (r = 0.060), but the p-value (0.529) was not significant (Table 2).

**Table 1:** Thyroid status and PSS PSS: Perceived Stress Scale.

Variable	Frequency	Percentage
Thyroid status		
Normal	93	81.6
Subclinical	21	18.4
PSS score		
Low	20	17.5
Moderate	90	78.9
High	4	3.5

**Table 2:** Correlation of PSS with T3, T4, and TSH PSS: Perceived Stress Scale; TSH: thyroid-stimulating hormone; T3: total triiodothyronine; T4: total thyroxine.

Variable	r-Value	p-Value
PSS score with T3	0.023	0.807
PSS score with T4	-0.082	0.387
PSS score with TSH	0.060	0.529

Of the 114 participants, 85.1% were married and 14.9% were unmarried. Out of the 85.1%, 81.7% had normal TSH, and the remaining 3.4% were married women having subclinical hypothyroidism (SCH). When the participants were compared concerning their marital status, the "p" value was statistically significant (p = 0.034). The mean and standard deviation (SD) for the duration of married life were higher and statistically significant (p < 0.001) among women with SCH (14.9 ± 4.65) compared to women with normal thyroid function (9.54 ± 7.02) (Table 3).

**Table 3:** Profile comparison among normal and subclinical hypothyroid participants. BMI: body mass index; SBP: systolic blood pressure; DBP: diastolic blood pressure; T3: total triiodothyronine; T4: total thyroxine; TSH: thyroid-stimulating hormone; PSS: Perceived Stress Scale.

Variable	Total participants	Normal (n = 93)	SCH (n = 21)	p-Value
Thyroid status		93 (81.6%)	21 (18.4%)	
Age	34.2 ± 6.1	33.22 ± 6.06	38.38 ± 23.2	<0.001
BMI	35.9 ± 7.9	35.26 ± 7.85	38.7 ± 4.96	0.014
Pulse rate	83.2 ± 11.4	83.25 ± 11.53	83.14 ± 11.2	0.97
SBP	118 ± 19.3	116 ± 18.75	123.4 ± 20.8	0.127
DBP	76.8 ± 11.9	76.49 ± 11.45	78.2 ± 13.8	0.557
Married life	10.5 ± 6.9	9.54 ± 7.02	14.9 ± 4.65	0.001
T3	2.32 ± 1.8	2.36 ± 1.9	2.14 ± 0.4	0.607
T4	9.01 ± 1.8	9.2 ± 1.65	8.4 ± 2.2	0.064
TSH	3.69 ± 9.3	2.17 ± 0.81	10.4 ± 20.6	<0.001
Marital status				
Married	97 (85.1%)	76 (81.7%)	21 (100)	0.034
Unmarried	17 (14.9%)	17 (18.3%)	0(0)	
Education				
Graduate	37 (32.5%)	31 (33.3%)	6 (28.6%)	0.67
Postgraduate	77 (67.5%)	62 (66.7%)	15 (71.4%)	
PSS Score				
High	4 (3.5%)	2 (2.2%)	2 (9.5%)	1
Moderate	90 (78.9%)	73 (78.5%)	17 (81%)	0.06
Low	20 (17.5%)	18 (19.4%)	2 (9.5%)	0.025
Menstrual Phase				
Proliferative phase	63 (55.3%)	52 (82.5)	11 (17.5)	0.768
Secretory phase	51 (54.7%)	41 (80.4%)	10 (19.6%)	1

The mean and SD for BMI (38.7 ± 4.96) were higher among women with SCH, which were statistically significant (p = 0.014) compared to normal women (35.26 ± 7.85). The mean age (38.38 ± 23.2) of women having SCH was higher and statistically significant compared to the normal women (33.22 ± 6.06). The mean and SD (10.4 ± 20.6) for TSH levels among women having SCH were higher and statistically significant (p < 0.001) compared to the mean and SD (2.17 ± 0.81) of TSH levels among normal women. When PSS scores were compared between SCH and normal thyroid profile women, more moderate PSS scores were observed among SCH women (81%) when compared to women with normal thyroid profile (78.5), which were statistically not significant (p = 0.06). Low PSS scores were found in 19.4% of women with normal thyroid profiles compared to 9.5% of women with SCH, which were statistically significant (p = 0.025) (Table 3). Women in the secretory phase have 1.15 times higher odds of perceiving stress when compared to women in the proliferative phase, but this was statistically insignificant (p = 0.769) (Table 4).

**Table 4:** Binary logistic regression for the phase of the menstrual cycle SE: standard error.

Parameter	SE	p-Value	Odds ratio
Secretory versus proliferative phase	0.484	0.769	1.153

## Discussion

The present study was conducted in 114 apparently normal women of reproductive age to determine the relationship between perceived stress and thyroid function. Among the total participants, 81.6% had normal thyroid function as the thyroid parameters were in the normal reference range (serum TSH: 0.27-4.2 microIU/mL, total T3: 1.3-3.1 nmol/L, total T4: 5.13-14.06 micrograms per liter) and 18.4% had high serum TSH levels (>4.2 microIU/mL) with normal total T3 and total T4 levels. Hence, such participants were categorized as having SCH, as proved by other studies [12,13].

Of the participants, 17.5% had a low PSS score, 78.9% had a moderate PSS score, and 3.5% had a high perceived PSS score. A positive correlation was observed between perceived stress and TSH (r-value = 0.060). A positive relationship between TSH and cortisol has been shown by Dayan CM and Panicker V study [14]. The possible explanation for this is the subtle metabolic stress associated with hypothyroidism, whether clinical or subclinical. Metabolic stress stimulates the HPA axis, leading to increased production and release of adrenocorticotropic hormone (ACTH) by the pituitary gland, which in turn prompts the adrenal glands to produce and secrete the stress hormone cortisol [15]. As demonstrated by a study carried out by Hennessey JV and Espaillat R, our study also revealed a significantly higher age (p < 0.01) and BMI (p = 0.014) among women with SCH compared to women with normal thyroid function. Leptin produced by adipose tissue plays an important role in regulating thyroid hormones by stimulating the synthesis of TSH, which then increases TSH levels [16].

The pulse rate and blood pressure of women with normal thyroid function and women having SCH were similar. These observations were similar to those of Duntas LH, Chiovato L, and the findings of Hayashi T et al. in their study [17,18]. There was no significant difference between the two groups (normal thyroid function and SCH) concerning their educational levels. This is similar to the results of Mollehave, Tang L, et al., who did their study on the effect of level of education on incident testing and treatment for SCH and did not find any difference between education and hypothyroidism [19]. The dietary habits of both groups were almost the same, and the effect of dietary nutrients on thyroid function can be excluded. These observations are similar to those of Chaudhuri A and Koner S in their study [1].

Significantly higher TSH levels (p < 0.01) were observed among women having SCH compared to women with normal thyroid function. Also, a significant difference was observed between women having SCH and women with normal thyroid function for their marital status (p: 0.034) and married life (p: 0.001). Hence, being married is linked to a higher likelihood of having SCH, possibly due to increased stress associated with married life. These observations were consistent with the observations of Zhan L, et al. in their study [20]. Binary logistic regression for the phase of the menstrual cycle (secretory and proliferative) showed an odds ratio of 1.53, indicating that women in the secretory phase perceived a greater amount of stress, which is similar to the findings of Jain P, et al. in their study. Increased perceived stress during the luteal phase can be attributed to high sympathetic activity caused by reduced estrogen and raised progesterone levels affecting the hypothalamic-pituitary-ovary axis. This may also cause altered neurotransmitters and other brain functions [21].

Limitations of the study

A psychological assessment of the participants was not done, as hypothyroidism is usually associated with depression. Cortisol levels in participants were not measured, so the cause-effect relationship cannot be established in this study. Since the p-value for the correlation between perceived stress and thyroid function was insignificant in this study, further research is needed in this direction with a larger sample size to confirm the findings and generalize the results. Hyperprolactinemia is usually associated with hypothyroidism, and perceived stress is a significant contributing factor to it in women of reproductive age. However, the serum prolactin levels were not measured in this study.

## Conclusions

This study explored the intricate relationship between perceived stress and thyroid function among women within the reproductive age group who appeared to be healthy. The findings indicate a trend toward a positive correlation between PSS score and TSH levels, although this trend did not achieve statistical significance. Notably, the study identified a higher occurrence of SCH among women with elevated age, BMI, and length of married life. These factors were associated with higher levels of TSH, suggesting a predisposition to SCH. The research adds to understanding how lifestyle factors and physiological stress may influence endocrine function. It underscores the importance of considering these elements in managing and preventing thyroid disorders. For women in their reproductive years, especially those experiencing high stress levels, regular thyroid function monitoring may be beneficial as part of a comprehensive health management strategy.
